# Supplementary material for: Complexome profiling of the Chlamydomonas psb28 mutant reveals TEF5 as an early PSII assembly factor
Source: Plant Cell. 2025 Mar 17;37(6):koaf055. doi: 10.1093/plcell/koaf055 (PMC12164590; doi:10.1093/plcell/koaf055)
Supplement: koaf055_Supplementary_Data [file koaf055_supplementary_data.zip › psb28_interactive.zip!/plots/intensity/qualityPlots/peptides/PSAL_Cre12.g486300.t1.2.html]

### Cre12.g486300.t1.2

Trivial name: PSAL  
SeqCoverage [median]: 34.70  
Mass: 20.28 kDa  
  
 

| Sequence | MissedCleavage | Mass [Da] | wtIntensity | muIntensity | Score |
| --- | --- | --- | --- | --- | --- |
| AQVISPVNGDPFVGMLETPVTSAPIVATYLSNLPAYR | 0 | 3887.02 | 3874.05 | 0.00 | 60.22 |
| GVEIGLAHGFLLAGPFIK | 0 | 1838.05 | 395507.02 | 374424.00 | 168.50 |
| GVEIGLAHGFLLAGPFIKLGPLR | 1 | 2374.39 | 2241.26 | 0.00 | 66.33 |
| TGVAPVLR | 0 | 811.49 | 1218244.39 | 1090575.14 | 158.36 |
|  |  |  | 1619866.7 | 1464999.1 |  |
